# Human expression patterns: qualitative and quantitative analysis of thrombospondin‐1 under physiological and pathological conditions

**DOI:** 10.1111/jcmm.13565

**Published:** 2018-02-14

**Authors:** Chen Zhao, Jeffrey S. Isenberg, Aleksander S. Popel

**Affiliations:** ^1^ Department of Biomedical Engineering School of Medicine Johns Hopkins University Baltimore MD USA; ^2^ Division of Pulmonary, Allergy and Critical Care Department of Medicine Heart, Lung, Blood and Vascular Medicine Institute University of Pittsburgh School of Medicine Pittsburgh PA USA

**Keywords:** angiogenesis, cancer, cardiovascular disease, matricellular, peripheral arterial disease, thrombospondin‐1

## Abstract

Thrombospondin‐1 (TSP‐1), a matricellular protein and one of the first endogenous anti‐angiogenic molecules identified, has long been considered a potent modulator of human diseases. While the therapeutic effect of TSP‐1 to suppress cancer was investigated in both research and clinical settings, the mechanisms of how TSP‐1 is regulated in cancer remain elusive, and the scientific answers to the question of whether TSP‐1 expressions can be utilized as diagnostic or prognostic marker for patients with cancer are largely inconsistent. Moreover, TSP‐1 plays crucial functions in angiogenesis, inflammation and tissue remodelling, which are essential biological processes in the progression of many cardiovascular diseases, and therefore, its dysregulated expressions in such conditions may have therapeutic significance. Herein, we critically analysed the literature pertaining to TSP‐1 expression in circulating blood and pathological tissues in various types of cancer as well as cardiovascular and inflammation‐related diseases in humans. We compare the secretion rates of TSP‐1 by different cancer and non‐cancer cells and discuss the potential connection between the expression changes of TSP‐1 and vascular endothelial growth factor (VEGF) observed in patients with cancer. Moreover, the pattern and emerging significance of TSP‐1 profiles in cardiovascular disease, such as peripheral arterial disease, diabetes and other related non‐cancer disorders, are highlighted. The analysis of published TSP‐1 data presented in this review may have implications for the future exploration of novel TSP‐1‐based treatment strategies for cancer and cardiovascular‐related diseases.

**List of Main Topics**

- Thrombospondin‐1 (TSP‐1), a matricellular protein, is often induced at sites of injury and tissue remodelling, and it is secreted by circulating, stromal and parenchymal cells at very different quantities.
- The major cell‐surface receptors for TSP‐1, namely CD36, CD47 and integrins, participate in important cellular processes including apoptosis, angiogenesis, blood flow, phagocytosis, migration and immune regulation.
- TSP‐1 is known to inhibit angiogenesis and endothelial cell survival.
- Cancer patient data on TSP‐1 in blood and cancerous tissues suggest that the patterns of TSP‐1 expression compared to non‐cancer controls are highly heterogeneous across different cancer types and that the correlations between TSP‐1 levels in patients and survival are also cancer type‐specific.
- Human TSP‐1 protein expression in circulating blood is uniformly up‐regulated in a variety of cardiovascular and inflammatory diseases and is often associated with worse patient outcomes.

## 1 INTRODUCTION

Complex multicellular organisms require surface adhesion and a three‐dimensional support system. The extracellular matrix provides these necessary biomechanical structural elements.^1^ The generation and maintenance of this enveloping structural system are cooperatively regulated by a variety of cellular activities and signals. A specialized group of secreted proteins interface with and bind to the matrix and the cells contained by the matrix to act as intermediates to alter cell response primarily from outside‐in. This class of proteins has been termed matricellular proteins, and it represents an expanding group of molecules that includes over nine subfamilies of proteins that are increasingly recognized as playing important roles in homoeostasis and recently in diseases.^2^ As the name implies matricellular proteins function to span the area between the cell surface and the matrix scaffold. Also important to the understanding of these molecules is that they do not impart strength to the matrix themselves, instead they can regulate matrix metabolism to alter the biomechanical properties of the extracellular matrix.

**Figure 1:**
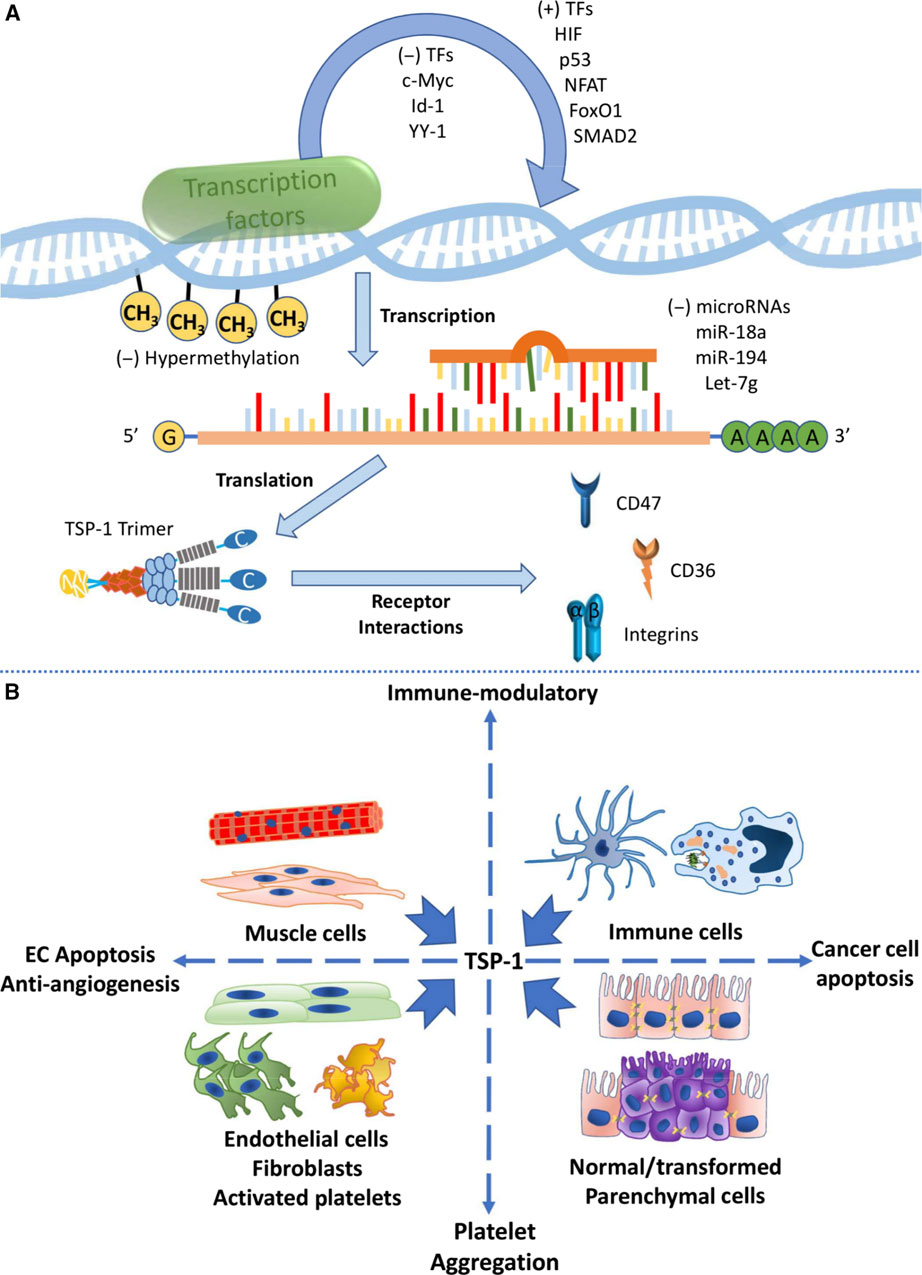
Regulation of thrombospondin‐1 (TSP‐1) at multiple levels and TSP‐1 secretion by different cells. (A) TSP‐1 gene transcription is regulated by multiple transcription factors and gene methylation status; TSP‐1 mRNA can be targeted by several microRNAs, and TSP‐1 protein which usually exists in trimers interacts with several cell‐surface receptors. (B) Various types of cells in humans produce and secrete TSP‐1, which can potently regulate many important cellular processes. Note that not all TSP‐1 receptors and functions are depicted here (see Conclusions and Future Considerations for more details)

A quintessential and founding member of this group is thrombospondin‐1 (TSP‐1), a protein first identified in the particulate fraction of thrombin‐activated platelets^3^ and this fact being incorporated in its name. Like many secreted proteins post‐development, TSP‐1 is minimally detectable in health but rapidly up‐regulated with injury and persists in chronic diseases, being found in the parenchyma as well as fluid compartments including the blood,^4^ urine^5^ and cerebrospinal fluid.^6^ TSP‐1 is trimeric, with each monomer about 130‐150 kD, but the secreted protein is heavily modified by glycosylation and weighs over 450 kD (Figure 1).^7^ Secreted TSP‐1 directly transduces signals through binding via discrete domains to cell‐surface receptors including but not limited to CD47, CD36 and integrins.^8^ Indirectly, TSP‐1 regulates cell signalling through binding to other molecules such as enzymes and growth factors.^9^, ^10^ Consequently, the manifold roles of TSP‐1 in modulating cell functions are concentration‐ and cell type‐specific. Nonetheless, some trends have emerged. From the perspective of pharmacology, high concentrations of TSP‐1 check the cell cycle in primary cells to impede self‐renewal and proliferation^11^ and can, at certain concentrations, induce cell death.^12^, ^13^ These effects arise, in part, from the ability of TSP‐1 to limit pro‐growth signals and several key elements of metabolism.^14^ Another essential feature of TSP‐1 is its ability to control tissue repair and remodelling in response to injury and stress, a property enhanced by its important function to increase transforming growth factor beta (TGF‐beta) activity.^15^ In the central nervous system, TSP‐1 is expressed and secreted by astrocytes and is a promoter of synapse formation as well as neuronal proliferation and differentiation.^16^ During immune activation, TSP‐1 has a supportive role and can increase the activation of inflammatory cells including monocytes,^17^ dendritic cells,^18^ macrophages^19^ and T cells.^20^ Conversely, in other settings such as during the resolution of inflammation, TSP‐1 may act to suppress inflammation.^21^, ^22^ A fundamental and well‐established property of TSP‐1 is to limit endothelial cell (EC)‐mediated angiogenesis by inhibiting the activity of vascular endothelial growth factor (VEGF)^23^ and the pleiotropic signals of the gasotransmitter nitric oxide (NO).^24^ In this way, TSP‐1 adversely impacts angiogenesis and blood flow^25^ and modulates blood pressure by limiting NO‐mediated vasorelaxation.^26^ In this latter capacity, TSP‐1 is now recognized to alter cardiovascular responses in general.^25^, ^27^ In the light of its role to retard angiogenesis, TSP‐1 is shown to suppress tumour growth and is often found down‐regulated in the tumour microenvironment coincident with accelerated tumour invasiveness.^28^ Increases in circulating TSP‐1 expression have also been found to be positively correlated with patient survival in some cancers.^29^, ^30^ Expectantly, TSP‐1‐derived drugs have been developed in the interest of inhibiting angiogenesis and treating cancer.^31^, ^32^ However, the effect of TSP‐1 in cancer is not simple, and it has been reported that TSP‐1 can also support tumour growth and spread.^33^ Interest has been growing in TSP‐1 as a possible biomarker and an important contributor to human diseases, particularly in age‐related and metabolic diseases.^34^, ^35^ At the same time, TSP‐1 and several of its cell‐surface receptors, notably CD36 and CD47, have and continue to be pursued as therapeutic targets.^36^, ^37^

Renewed appreciation of TSP‐1 in the pathophysiology of diseases has encouraged research into the expression of this protein in different cells and body compartments. However, a systematic characterization and quantification of published TSP‐1 expression data in diseases, both amount and rate of production, has not been previously approached. Such an analysis is important, and it can provide insight into the sometimes conflicting activity of this molecule and serve to guide therapeutic interventions that target pathways mediated by TSP‐1; it is also important for systems biology computational studies of TSP‐1.^38^, ^39^, ^40^ Herein, we present the results of a systematic multilevel (cell, fluid, tissue) characterization of TSP‐1 concentrations in people in health and disease, specifically in cancer and cardiovascular diseases, derived from analysis of the current literature. Interestingly, strong patterns emerge. In cancer, TSP‐1 expression is surprisingly heterogeneous to the point of forestalling prediction as to its role in many of these cases. Conversely, in inflammatory and cardiovascular diseases, TSP‐1 expression levels show a persistent trend to be significantly elevated compared to non‐diseased subjects. The correlation between dysregulated TSP‐1 expressions in diseases and patient outcomes is also discussed. These results emphasize the role of the protein, beyond the sphere of cancer, as indicative of promoting disease and as a possible therapeutic index.

## 2 TSP‐1 SECRETION FROM PARENCHYMA AND STROMA

Thrombospondin‐1 is reported to be expressed and secreted by a variety of normal cell types in human including ECs, fibroblasts, muscle cells, immune cells, platelets (and megakaryocytes) as well as transformed parenchymal cells in many types of cancer (Figure 1).^41^, ^42^, ^43^ Secreted TSP‐1 proteins play key roles in the regulation of angiogenesis and immune response, both of which are critical processes in the progression of tumours and cardiovascular diseases. However, specific cell types exist in quite different numbers within the whole tissue. Therefore, a direct, quantitative comparison of the absolute TSP‐1 secretion rates by different parenchymal and stromal cells will introduce new insight into the research of heterogeneity in the tumour microenvironment and other diseases.

Beginning at the cell level, a literature search was conducted and TSP‐1 secretion rates from different types of cells were analysed (Table 1).^44^, ^45^, ^46^, ^47^, ^48^, ^49^, ^50^, ^51^, ^52^, ^53^, ^54^ Interestingly, secretion rates of TSP‐1 protein from the stromal components (eg fibroblasts, ECs) are at least one to two orders of magnitude greater than those rates from cancer cells. Among different types of stromal cells, ECs (represented by human umbilical vein ECs) produce and secrete TSP‐1 proteins at very high rates. ECs usually occupy a fairly small portion (1%‐2%) of the total cells in a tissue, but this percentage can be significantly higher in some highly vascularized tissues such as in certain tumours, lungs and hearts.^55^, ^56^ In this sense, modulating EC‐specific pathways (eg via transcription factors, receptor activation and microRNAs) that regulate TSP‐1 production and secretion may substantially influence the overall TSP‐1 abundance in the tissue environment and vasculature. In contrast, the many types of cancer cells examined, except for glioma, Kaposi's sarcoma and prostate cancer cells, contribute relatively insignificant amounts of TSP‐1 on a rate per million cells basis. Analyses of tumour sample immunohistochemical staining confirm the high TSP‐1 expression and localization in the tumour stroma rather than in the tumour cells.^44^, ^57^, ^58^ In addition, published data of TSP‐1 mRNA expressions across various cell lines show a qualitatively similar trend compared with the secretion rates presented in Table 1.^59^ Still, production and secretion rates of TSP‐1 would depend on various factors including the density of cultured cells and also the appropriate stimuli which are present in tissue environments in vivo but may not be contained in the culture media; therefore, the differences between cell‐specific secretion rates outlined here should be interpreted in both quantitative and qualitative manners. Further studies and measurements are needed to elucidate the potential correlations between in vitro (summarized in this review) and in vivo TSP‐1 production capacities in the different cell types.

**Table 1:** TSP‐1 secretion rates from different cancer and non‐cancer cell types

Human cell type (cell line)	TSP‐1 protein secretion rate (ng/10^6^ cells/24 h)	References
Pancreatic cancer (AsPC‐1; Colo‐357; Panc‐1; T3M4)	276; 61; 90; 94	44
Glioma (T98G; U251; A172; KG‐1‐C; TM2; YMG1; YMG2; YMG3; YMG4; YMG5)	2431; 275; 59; 43; 475; 69; 1081; 1450; 126; 250	45
Breast cancer (YMB‐1)	3	45
Breast cancer (T47D; BT‐474)^a^	3; 3	46
Lung cancer (A549)	20	45
Gastric cancer (NUGC‐4)	31	45
Hepatic cancer (HLF)	89	45
Colon cancer (Colo‐201)	3	45
Prostate cancer (PC3)	610	45
Melanoma (DFB)	8	45
Neuroblastoma (IMR‐32)	4	45
B‐Chronic lymphocytic leukaemia (B cells from patients)	9	47
Promyelocytic leukaemia (NB4; HL‐60)^b^	55; 40	48
Kaposi's sarcoma (IST‐KS XVI; IST‐KS VIII; IST‐KS XI; IST‐KS IV)	6500; 3400; 4400; 8500	51
Human foreskin fibroblast	474	44
Human foreskin fibroblast^c^	15 700	49
Human foreskin fibroblast^c^	3333	50
Human foetal lung fibroblast (GM1604)^c^	5800	49
Endothelial cell (HUVEC)^c^	21 000	49
Endothelial cell (HUVEC)	19 500	51
Endothelial cell (HUVEC)^c^	49 000	52
Human aortic smooth muscle cell^c^	9467	50
Human dendritic cell^d^	10 153; 3053	53
Human retinal glial cell (MIO‐M1)	125	54

a Unit conversion is implemented by assuming 150 pg of total protein per cell.^60^

b Cells are treated with all‐trans retinoid acid.

c Study did not specify that TSP data are limited to TSP‐1.

d Cells are treated with ATP and prostaglandin E2, respectively. Values are rounded to the nearest integer.

## 3 QUANTITATIVE TSP‐1 EXPRESSION PROFILES IN PATHOLOGICAL CONDITIONS

### 3.1 Human cancers

The potential of circulating and tissue TSP‐1 protein as diagnostic or prognostic markers for cancers, given its anti‐angiogenic and pro‐apoptotic properties, has been studied extensively. Table 2 summarizes the quantitatively measured TSP‐1 protein levels in the plasma, serum, platelet and tissue of individuals with various cancer conditions.^61^, ^62^, ^63^, ^64^, ^65^, ^66^, ^67^, ^68^, ^69^, ^70^, ^71^, ^72^, ^73^, ^74^, ^75^, ^76^, ^77^, ^78^, ^79^, ^80^, ^81^, ^82^, ^83^, ^84^, ^85^ Most measured values of plasma TSP‐1 protein levels are in the range of a few hundred to a few thousand ng/mL, indicating that physiological TSP‐1 concentrations are relatively low in the circulation.^86^ It should be noted that data on soluble plasma and serum TSP‐1 may be confounded by varying degrees of platelet activation that serve as a reservoir of pre‐formed TSP‐1 in alpha granules during sample acquisition and processing.^42^ Nonetheless, it is worth noting that a large portion of the plasma TSP‐1 data actually indicate an up‐regulation of TSP‐1 in patients with cancer compared to normal controls, especially in breast cancer, which might be considered counterintuitive to the well‐established anti‐angiogenic property of TSP‐1. In the case of breast cancer (general disease and not in the context of any specific subtypes), four separate studies have found a consistent, significant increase in plasma or tissue TSP‐1 protein in patients with cancer compared to healthy controls.^67^, ^71^, ^83^, ^87^ A strong positive correlation between plasma and intratumoural TSP‐1 is observed, and patients with lymph node metastasis have significantly higher plasma TSP‐1 compared to lymph node‐negative patients. Intratumoural TSP‐1 expression is also positively correlated with microvessel density, suggesting a pro‐angiogenic role of TSP‐1 in breast cancer.^67^ The up‐regulation of tissue TSP‐1 expression in breast cancer is further supported by TSP‐1 mRNA and immunohistochemical data.^88^, ^89^ In non‐small cell lung cancers, significantly lower plasma and serum levels of TSP‐1 have been observed in patients,^75^ and higher baseline serum TSP‐1 levels are found associated with increased overall survival in patients receiving treatments.^76^ In colon cancer, conflicting results of plasma TSP‐1 in patients *versus* controls have been reported.^72^, ^73^ In pancreatic cancer, serum TSP‐1 is down‐regulated in patients with cancer.^78^, ^79^ In glioblastoma, no significant difference is observed in serum TSP‐1 levels in patients compared to healthy subjects, but higher pre‐surgery serum TSP‐1 is prognostic of longer survival in patients after tumour resection.^80^ Overall, these data suggest that the cancer‐driven regulation of TSP‐1 expression in humans may be highly dependent on the specific cancer types and clinical stage and deserves further investigation. Given the high TSP‐1 secretion rates in stromal cells, the potential for cancer cells to up‐ and down‐regulate stromal cell TSP‐1 production to favour cancer growth and metastasis should also be considered in future TSP‐1 studies.

**Table 2:** Circulating and tissue TSP‐1 protein levels in healthy (control) versus cancer subjects

Cancer type studied	Plasma TSP‐1 (control) (ng/mL)	Plasma TSP‐1 (patient) (ng/mL)	*P* value of difference	No. of subjects (C; P)	References
Mixed	N/A	54 (R = 7‐551)	N/A	N/A; 50	61
Mixed	399 (SEM = 61)	491 (SEM = 66)	.3	43; 43	62
**Mixed** a	**440 (IQR = 270‐559)**	**850 (IQR = 493‐1336)**	**<.01**	**20; 24**	**[** 63 **]**
**Mixed**	**31 (IQR = 25‐34)**	**73 (IQR = 34‐84)**	**<.001**	**12; 20**	**[** 64 **]**
**AML** a	**121 (IQR = 65‐181)**	**11 (IQR = 7‐15)**	**<.01**	**12; 17**	**[** 65 **]**
GI, breast, lung^a^	365	1095, 730, 1095	N/A	20; (22, 18, 17)	66
**EBC, ABC**	**221 (IQR = 175‐247)**	**484, 588 (IQR = 344‐877, 430‐952)**	**<.05, <.001**	**36; (71, 66)**	**[** 67 **]**
Breast	N/A	280 (SEM = 53)	N/A	N/A; 12	68
Breast	396 (SD = 103)	419 (SD = 102)	.45	65; 37	69
Breast (metastatic)	543 (IQR = 504‐967)	2255 (IQR = 681‐4553)	.07	16;8	70
**Breast** a	**190 (SD = 42)**	**2482 (SD = 4095)**	**<.0001**	**31; 23**	**[** 71 **]**
**Colon (dukes stage** A**, B, C, D)** a	**124 (SD = 63)**	286**, 389, 781, 1017 (SD = **211**, 234, 589, 668)**	**<.05 for Stage B, C, D**	**20; (**42**, 24, 21, 28)**	**[** 72 **]**
**Colon**	**1698 (IQR = 1437‐2703)**	**328**	**<.001**	**36; 33**	**[** 73 **]**
Colon	539 (SD = 389)	412 (SD = 367)	NS	84; 35	74
**NSCLC**	**4167 (IQR = 3585‐5472)**	**2500**	**.004**	**46; 21**	**[** 75 **]**

Cancer type studied	Serum TSP‐1 (control) (μg/mL)	Serum TSP‐1 (patient) (μg/mL)	*P* value of difference	No. of subjects (C; P)	References
**NSCLC**	**108 (IQR = 49‐225)**	**35**	**.012**	**46; 21**	**[** 75 **]**
NSCLC	48 (SD = 10)	45 (SD = 16)	.3	60; 60	76
NSCLC	180 (R = 110‐201)	177 (R = 97‐206)	.158	18; 40	77
**Pancreatic**	**11**	**8**	**<.0001**	**30; 34**	**[** 78 **]**
**Pancreatic** a	**16**	**14**	**<.01**	**227; 333**	**[** 79 **]**
Glioblastoma	67 (SD = 37)	78 (SD = 26)	.37	9; 23	80
HCC	N/A	17 (IQR = 11‐23)	N/A	N/A; 60	81

Cancer type studied	Platelet TSP‐1 (control) (ng/10^6^ platelets)	Platelet TSP‐1 (patient) (ng/10^6^ platelets)	*P* value of difference	No. of subjects (C; P)	References
Breast	27 (IQR = 24‐59)	29 (IQR = 22‐78)	.821	65; 37	69
NSCLC	9 (IQR = 2‐22)	15 (IQR = 3‐58)	.092	68; 68	82
Colon	34 (R = 14‐100)	35 (R = 17‐139)	.88	84; 35	74
**Mixed**	**38 (IQR = 28‐45)**	**29 (IQR = 18‐38)**	**.005**	**43; 43**	**[** 62 **]**

Cancer type studied	Tissue TSP‐1 (control/benign) (μg/g of total protein)	Tissue TSP‐1 (patient/cancer) (μg/g of total protein)	*P* value of difference	No. of subjects (C; P)	References
**Breast** a	**22**	**317**	**<.000001**	**15; 101**	**[** 83 **]**
Breast^a^	N/A	6 (IQR = 4‐10)	N/A	N/A; 166	84
**Adrenocortical**	**142 (R = 40‐390)**	**69 (R = 8‐344)**	**<.01**	**18; 13**	**[** 85 **]**
HCC	N/A	9 (IQR = 5‐16)	N/A	N/A; 60	81

Mean/median values are shown in the left of the 2nd and 3rd columns; variations of measurements are shown in the right. Statistical analysis results are shown in the 4th column.

R, range; SEM, standard error of the mean; IQR, interquartile range; SD, standard deviation; N/A, not applicable; NS, non‐significant; AML, acute myeloid leukaemia; GI, gastrointestinal; EBC, early breast cancer; ABC, advanced breast cancer; NSCLC, non‐small cell lung cancer; HCC, hepatocellular carcinoma.

a Study did not specify that TSP data are limited to TSP‐1. Values are rounded to the nearest integer. Rows with *P* values smaller than .05 (indicating statistical significance of the difference observed) are bolded.

### 3.2 Cardiovascular diseases

In contrast to cancer, angiogenesis is often impaired and therefore desired in many age‐related and cardiovascular diseases, especially in ischaemic vascular diseases such as coronary artery disease (CAD) and peripheral arterial disease (PAD). One hypothesis offered to explain the pathophysiology of CAD and PAD is that anti‐angiogenic factors (eg TSP‐1) may be highly up‐regulated in the ischaemic tissue, in addition to the insufficient induction of pro‐angiogenic factors (eg VEGF, NO).^39^, ^90^ To date, only a few studies have explored this hypothesis and confirmed the increase in plasma and skeletal muscle TSP‐1 in PAD^91^, ^92^, ^93^ and CAD^94^ patients. Related to this, blockade of TSP‐1/CD47 signalling can enhance ischaemic tissue survival in experimental PAD models.^95^ Further, TSP‐1 protein levels in the plasma are significantly elevated in patients who suffer from other cardiovascular and inflammatory diseases, as well as diseases that are commonly accompanied by cardiovascular complications,^96^ including diabetes^97^ and sickle cell disease^4^, ^98^ (Table 3). Marked up‐regulations of TSP‐1 have been observed in the various organs and tissues of patients with diabetes and also in animal models of diabetes.^99^ This may be in part secondary to the known effects of high glucose on TSP‐1 production.^100^ In terms of disease outcome, strong negative correlations between plasma TSP‐1 protein levels and patient survival have been observed for pulmonary hypertension, acute ischaemic stroke and end‐stage renal disease, all conditions characterized by vasculopathy.^101^, ^102^, ^103^ Interestingly, on the other hand, thrombospondin proteins including TSP‐1 are involved in the unfolded protein response (also known as the endoplasmic reticulum stress response), and they are found to be induced and exert protective effects following myocardial injury in animal models, which adds another layer of complexity to the functions of the up‐regulated TSP‐1 in cardiovascular diseases.^104^

**Table 3:** Circulating and tissue TSP‐1 levels in healthy (control) versus cardiovascular/CV‐related disease subjects

CV‐related disease studied	Plasma TSP‐1 (control) (ng/mL)	Plasma TSP‐1 (patient) (ng/mL)	*P* value of difference	No. of subjects (C; P)	References
**PAD**	**218**	**476**	**<.0001**	**184; 330**	**[** 91 **]**
PAD	176 (SEM = 58)	160 (SEM = 62)	NS	17; 17	92
**Pulmonary hypertension**	**82 (SD = 16)**	**1114 (SD = 136)**	**<.05**	**19; 93**	**[** 101 **]**
Sickle cell disease	239 (IQR = 125‐344)	303 (IQR = 187‐939)	.056	17; 27	4
Sickle cell disease	491 (R = 331‐723)	536 (R = 333‐1107)	NS	8; 14	98
**Ischaemic stroke**	**146 (SD = 50)**	**571 (SD = 226)**	**<.001**	**150; 192**	**[** 102 **]**
**Type I diabetes** a	**91 (SEM = 14)**	**137 (SEM = 14)**	**<.05**	**15; 30**	**[** 97 **]**
**Vasculitis** a	**59 (SD = 29)**	**791 (SD = 1412)**	**.0002**	**33; 20**	**[** 96 **]**
**CAD and DM**	**518 (SD = 127)**	**579 (SD = 106)**	**<.01**	**108; 103**	**[** 94 **]**

CV‐related disease studied	Interstitial TSP‐1 (control) (ng/mL)	Interstitial TSP‐1 (patient) (ng/mL)	*P* value of difference	No. of subjects (C; P)	References
**PAD (muscle dialysate)**	**54 (SEM = 24)**	**219 (SEM = 70)**	**<.05**	**7; 6**	**[** 92 **]**
Healthy (muscle dialysate)	100 (SEM = 18)	N/A	N/A	8; N/A	132

CV‐related disease studied	Proximal/healthy tissue (AU)	Distal/ischaemic tissue (AU)	*P* value of difference	No. of subjects (C; P)	References
**Tissue TSP‐1 mRNA in amputated limbs from CLI patients**	**2 (SEM = 0.8)**	**21 (SEM = 3.9)**	**.0001**	**13**	**[** 93 **]**

Mean/median values are shown in the left of the 2nd and 3rd columns; variations of measurements are shown in the right. Statistical analysis results are shown in the 4th column.

R, range; SEM, standard error of the mean; IQR, interquartile range; SD, standard deviation; N/A, not applicable; NS, non‐significant; DM, diabetes mellitus; CLI, critical limb ischaemia; AU, arbitrary units.

a Study did not specify that TSP data are limited to TSP‐1. Values are rounded to the nearest integer (except for values less than 1). Values are for TSP‐1 protein levels unless otherwise noted. Rows with *P* values smaller than .05 (indicating statistical significance of the difference observed) are bolded.

### 3.3 Correlations between TSP‐1 and VEGF

Paired data on quantitative TSP‐1 and VEGF protein expressions in cancers and PAD are shown in Table 4. While the trend of TSP‐1 expression in cancers remains elusive, VEGF levels (plasma, serum, platelet, tissue) tend to be up‐regulated in most cases.^105^ Published data so far have not suggested any correlation between circulating levels of VEGF and TSP‐1 in patients with cancer (Table 4), but elevated circulating VEGF expression alone is a well‐established prognostic marker of decreased patient survival in several types of cancer.^106^ Some previous studies have tried to elucidate the potential relationship between VEGF and TSP‐1 expressions within tumours. Although inverse correlation between tumour VEGF and TSP‐1 expressions has been suggested in prostate and endometrial cancers,^57^, ^107^ it may not always be the case, at least in bladder cancer, gastric cancer and hepatocellular carcinoma patients in which TSP‐1 protein levels are shown to be positively correlated with VEGF protein levels in the tumour tissue.^81^, ^108^, ^109^ In PAD studies, so far no correlations between TSP‐1 and VEGF levels in patients have been suggested, although both proteins are found up‐regulated in the patient plasma, and muscle interstitial TSP‐1, but not VEGF, is significantly induced in PAD patients which may indicate a potential imbalance between pro‐angiogenic and anti‐angiogenic factors in the ischaemic tissue.^91^, ^92^, ^110^, ^111^ Feedforward and feedback interactions between VEGF and TSP‐1 through direct intersection and with the gasotransmitter NO likely also complicate these expression patterns.^23^, ^38^ Moreover, the interplay between VEGF and TSP‐1 exists not only in angiogenic pathways, as both VEGF and TSP‐1 are shown to be regulators of the immune system.^19^, ^20^, ^112^

**Table 4:** Circulating and tissue TSP‐1/VEGF protein levels in healthy (control) versus disease subjects

Disease studied	Plasma TSP‐1 (control, patient) (ng/mL)	Plasma VEGF (control, patient) (pg/mL)	No. of subjects (C; P)	References
Cancer (mixed)	399, 491 NS	**7, 44** ***P*** ** = .003**	43; 43	62
Breast cancer	396, 419 NS	53, 54 NS	65; 37	69
Breast cancer (metastatic)	543, 2255 NS	**12, 29** ***P*** ** = .001**	16; 17	70
Colon cancer	**1698, 328** ***P*** ** < .001**	**2, 48** ***P*** ** < .001**	36; 33	73
Colon cancer	539, 412 NS	53, 40 NS	84; 35	74
PAD	**218, 476** ***P*** ** < .0001**	14, 17 NS	184; 330	91

Disease studied	Serum TSP‐1 (control, patient) (μg/mL)	Serum VEGF (control, patient) (pg/mL)	No. of subjects (C; P)	References
NSCLC	**108, 35** ***P*** ** = .012**	**249, 452** ***P*** ** < .001**	46; 21	75
NSCLC	48, 45 NS	**147, 408** ***P*** ** < .0001**	60; 60	76
NSCLC	180, 177 NS	189, 266 NS	18; 40	77

Disease studied	Platelet TSP‐1 (control, patient) (ng/10^6^ platelets)	Platelet VEGF (control, patient) (pg/10^6^ platelets)	No. of subjects (C; P)	References
Breast cancer	27, 29 NS	**0.9, 2** ***P*** ** < .001**	65; 37	69
NSCLC	9, 15 NS	**22, 41** ***P*** ** = .041**	68; 68	82
Colon cancer	34, 35 NS	**0.6, 1.3** ***P*** ** < .0001**	84; 35	74
Cancer (mixed)	**38, 29** ***P*** ** = .005**	**0.6, 1.4** ***P*** ** < .0001**	43; 43	62

Disease studied	Tissue TSP‐1 (control/benign, patient/cancer) (μg/g of total protein)	Tissue VEGF (control/benign, patient/cancer) (ng/g of total protein)	No. of subjects (C; P)	References
Adrenocortical cancer	**142, 69** ***P*** ** < .01**	**44, 404** ***P*** ** < .001**	(18; 13), (15; 12)	85

Disease studied	Interstitial TSP‐1 (control, patient) (ng/mL)	Interstitial VEGF (control, patient) (pg/mL)	No. of subjects (C; P)	References
PAD	**54, 219** ***P*** ** < .05**	55, 63 NS	(7; 6), (16; 16)	92

Mean/median values are shown in the left of the 2nd and 3rd columns; statistical analysis results are shown in the right. *P* values greater than .05 are denoted as NS (non‐significant), and observations with *P* < .05 are bolded. Values are rounded to the nearest integer (except for values less than 2).

## 4 CONCLUSIONS AND FUTURE CONSIDERATIONS

Herein, we systematically reviewed the literature characterizing human TSP‐1 expressions in the circulation and in tissues, its cell type‐specific secretion and its significance and correlations with outcomes in human diseases. This analysis is driven by quantitative data and demonstrated some interesting findings. In cancer, TSP‐1 expression patterns in general are quite variable and, thus, appear to have limited prognostic or diagnostic value, although in specific cancer types (eg breast cancer), TSP‐1 up‐regulation is relatively consistent across independent datasets and is associated with malignancy and metastasis. In addition, a similar paradox in expression patterns pertains to TSP‐1 and VEGF in general cancer conditions. Contrary to the situation in cancer, TSP‐1 expression is uniformly up‐regulated in cardiovascular and inflammatory diseases and is associated with worse outcomes, albeit the studies testing the latter hypothesis are of limited number. Interpretation of these results should be tempered as expression data may not distinguish between changes in TSP‐1 production/secretion and uptake/degradation/cleavage. Although TSP‐1 in blood could likely have an effect on circulating inflammatory cells, red blood cells and platelets to increase inflammation, adhesion and aggregation, since TSP‐1 binds to various components in the extracellular matrix, its up‐ and down‐regulations as measured in circulating blood may not reflect the actual changes of TSP‐1 abundance and its functional activities in the matrix. A further limitation is that very few quantitative data on tissue TSP‐1 in diseases are available in the literature, and it should be pointed out that the protein expression levels may not correlate with activation of downstream signalling. Finally, being obtained from human subjects, the majority of these expression data lack time course analysis that could contribute to a better understanding of the changes noted. These caveats aside, the literature data collected and presented in our quantitative analysis remain the first of their kind and should serve to guide future basic and translational research.

Specific domains of TSP‐1 interact with different proteins and non‐protein molecules in the extracellular matrix, and the affinity Kd values of these interactions are mostly in the nmol L^−1^ range.^9^, ^113^ The cell‐surface receptors that TSP‐1 interacts with also have important therapeutic value in cancer and cardiovascular diseases. CD36 is a low‐affinity receptor which recognizes the type I repeats of TSP‐1 and is reported to be an activator of the apoptotic pathways in ECs and some cancer cells.^114^, ^115^, ^116^ The first TSP‐1‐based therapeutic (ABT‐510) was developed based on this interaction more than a decade ago. However, it failed to demonstrate clinical efficacy against metastatic cancers and did not move forward beyond phase II trials.^117^, ^118^ Similar to ABT‐510, several other peptides that are derived from the type I repeats were shown to be anti‐angiogenic in vitro.^119^, ^120^ In recent years, CD47, a high‐affinity receptor which interacts with the C‐terminal domain of TSP‐1, has garnered attention.^121^ Accumulating evidence has suggested that the TSP‐1/CD47 axis could be a promising therapeutic target in cancer as well as in cardiovascular diseases.^32^, ^36^ Besides its well‐established anti‐angiogenic and anti‐proliferative effects when engaged with TSP‐1,^23^ CD47 also participates in immune suppression of macrophages and is found widely overexpressed in different types of cancer.^122^ As a self‐recognition signal, CD47 on cancer cells associates with SIRPα (signal regulatory protein alpha) on macrophages and inhibits phagocytic activities. Interestingly, TSP‐1 also interacts with SIRPα expressed on non‐phagocytic cells; however, the potential role of how TSP‐1 modulates the CD47/SIRPα axis during macrophage activation remains unclear.^32^, ^123^ Another class of TSP‐1 receptors is the integrins that recognize the RGD motifs (Arg‐Gly‐Asp) of TSP‐1,^124^ and intuitively, TSP‐1 can facilitate the adhesion of various cells, including cancer cells, to the extracellular matrix.^125^ Other major factors that TSP‐1 interacts with include heparin, CD148, syndecan‐1, calreticulin/LRP‐1 complex (low‐density lipoprotein receptor‐related protein 1), and it has also been suggested that TSP‐1 may trigger pro‐survival and pro‐migratory functions in cells through binding with some of its receptors.^9^, ^45^, ^126^ Indeed, few studies have tracked TSP‐1 protein expression concurrent with its receptor level expression. In terms of studying how TSP‐1‐mediated signal transduction contributes to diseases, it will be important to track both the ligand TSP‐1 as well as its specific receptors in cell and tissue compartments.

Besides diseases, many natural biological processes can also contribute to the endogenous regulation of TSP‐1. Preclinical studies in mice have demonstrated age‐associated up‐regulation of TSP‐1 in kidney,^127^ heart^128^ and skin.^129^ The fact that both TSP‐1 and CD47 are significantly induced in the skin of aged mice and negatively act on blood flow may imply a deleterious role of TSP‐1/CD47 axis in ageing and ageing‐related complications.^95^ In addition, diabetes‐induced up‐regulation of TSP‐1 may contribute to ageing‐related vascular rarefaction in the hearts of leptin‐resistant mice, and loss of TSP‐1 expression can attenuate this pattern.^130^ Exercise is another factor that seems to control TSP‐1 dynamics. Confirmed in both mice and humans, TSP‐1 expression is greatly increased in skeletal muscles following active training and this is accompanied by an increase in VEGF expression.^131^, ^132^ Interestingly, a delay is observed between TSP‐1 induction and VEGF induction (VEGF induction preceding TSP‐1), which suggests endogenous feedback mechanisms through timely regulation of pro‐ and anti‐angiogenic factors to sustain adequate but not excessive angiogenesis and blood flow during and after exercise.^133^ Gestation status can also affect TSP‐1 expression in the uterus, in which TSP‐1 expression was shown to increase over the last few weeks before labour and peak during labour,^134^ putatively where its role to promote platelet activation by resisting NO‐mediated effects on platelets^135^ and blood vessels^26^ may have beneficial effects to limit haemorrhage.

The inconsistent pattern of TSP‐1 expression observed in different types of cancer is reflected in the equally controversial role of tumour TSP‐1 expression as a survival predictor. In accordance to its anti‐angiogenic property, high tumour expression of TSP‐1 is correlated with increased patient survival in colon,^136^ lung,^137^ bladder,^138^ ovarian,^139^ cervical^140^ and gastric cancer.^109^ However, high tumour tissue TSP‐1 is also associated with decreased survival in patients with hepatocellular carcinoma,^81^ breast cancer^141^ and melanoma.^142^ Along with the observation that high VEGF in cancer is usually associated with worse prognosis and increased metastasis,^106^ it is very likely that certain types of cancer (eg breast cancer) may have developed compensatory mechanisms to counteract the anti‐angiogenic pathways activated by TSP‐1.^143^, ^144^ Resistance to apoptosis and increased VEGF secretion in response to the hypoxic environments resulting from the TSP‐1‐induced loss of tumour vascularization are possible explanations, which hints future investigations of combination therapies that target both molecules in cancer treatments. Separate from cancer, anti‐angiogenic molecules such as TSP‐1 and VEGF_165_b have emerged as new promising targets in ischaemic vascular diseases (eg PAD).^145^, ^146^ Therapeutics that inhibit TSP‐1 as well as its downstream pathways through small molecule/RNAi‐based inhibitors, modulation of upstream transcription factors and TSP‐1 receptor antibodies or morpholino oligonucleotides could potentially be novel modes to accelerate muscle perfusion and regeneration, given that single‐agent gene therapies that deliver pro‐angiogenic factors (eg VEGF, FGF, HGF) so far have been unsuccessful clinically.^147^ Conversely, in the scenario of age‐related macular degeneration where TSP‐1 levels in the eye are greatly reduced, enhancing TSP‐1 expression may help to counteract the excessive angiogenesis and restore the balance between pro‐ and anti‐angiogenic factors.^148^

## CONFLICT OF INTEREST

J.S.I. serves as Chair of the Scientific Advisory Board of Radiation Control Technologies, Inc. (RCTI, Garden City, NJ) and has equity interest in RCTI and Tioma Therapeutics (St. Louis, MO) that licensed CD47 technology for development. The other authors have declared that no competing interests exist.
